# Gene expression profiles of carotid plaques differ between patients with amaurosis fugax and those with cerebral transient ischaemic attack or stroke

**DOI:** 10.1093/cvr/cvag116

**Published:** 2026-06-02

**Authors:** Tian Lan, Floor B H van der Zalm, Kaylin C A Palm, Yayuan Zhu, Ernest Diez Benavente, Sander W van der Laan, Dominique P V de Kleijn, Ynte M Ruigrok, Joost A van Herwaarden, Gert J de Borst, Hendrik Bart van der Worp, Martin Dichgans, Hester M den Ruijter, Gerard Pasterkamp, Michal Mokry

**Affiliations:** Laboratory of Experimental Cardiology, Department of Cardiology, University Medical Center Utrecht, University Utrecht, Heidelberglaan 100, 3584 CX Utrecht, The Netherlands; Central Diagnostic Laboratory, Division Laboratories, Pharmacy, and Biomedical Genetics, University Medical Center Utrecht, University Utrecht, Heidelberglaan 100, 3584 CX Utrecht, The Netherlands; Central Diagnostic Laboratory, Division Laboratories, Pharmacy, and Biomedical Genetics, University Medical Center Utrecht, University Utrecht, Heidelberglaan 100, 3584 CX Utrecht, The Netherlands; Central Diagnostic Laboratory, Division Laboratories, Pharmacy, and Biomedical Genetics, University Medical Center Utrecht, University Utrecht, Heidelberglaan 100, 3584 CX Utrecht, The Netherlands; Laboratory of Experimental Cardiology, Department of Cardiology, University Medical Center Utrecht, University Utrecht, Heidelberglaan 100, 3584 CX Utrecht, The Netherlands; Central Diagnostic Laboratory, Division Laboratories, Pharmacy, and Biomedical Genetics, University Medical Center Utrecht, University Utrecht, Heidelberglaan 100, 3584 CX Utrecht, The Netherlands; Laboratory of Experimental Cardiology, Department of Cardiology, University Medical Center Utrecht, University Utrecht, Heidelberglaan 100, 3584 CX Utrecht, The Netherlands; Central Diagnostic Laboratory, Division Laboratories, Pharmacy, and Biomedical Genetics, University Medical Center Utrecht, University Utrecht, Heidelberglaan 100, 3584 CX Utrecht, The Netherlands; Department of Genome Sciences, University of Virginia, 1335 Lee Street, West Complex, Charlottesville, VA 22903, USA; Department of Vascular Surgery, University Medical Centre Utrecht, Heidelberglaan 100, 3584 CX Utrecht, The Netherlands; Department of Neurology and Neurosurgery, UMC Utrecht Brain Center, University Medical Center Utrecht, Heidelberglaan 100, 3584 CX Utrecht, The Netherlands; Department of Vascular Surgery, University Medical Centre Utrecht, Heidelberglaan 100, 3584 CX Utrecht, The Netherlands; Department of Vascular Surgery, University Medical Centre Utrecht, Heidelberglaan 100, 3584 CX Utrecht, The Netherlands; Board of Directors, Reinier de Graaf Gasthuis, Reinier de Graafweg 5, 2625 AD Delft, The Netherlands; Department of Neurology and Neurosurgery, UMC Utrecht Brain Center, University Medical Center Utrecht, Heidelberglaan 100, 3584 CX Utrecht, The Netherlands; Institute for Stroke and Dementia Research (ISD), University Hospital LMU, Feodor-Lynen-Straße 17, Munich 81377, Germany; Munich Cluster for Systems Neurology (SyNergy), Institute for Stroke and Dementia Research (ISD), Feodor-Lynen-Straße 17, Munich 81377, Germany; German Center for Neurodegenerative Diseases (DZNE) Munich, Institute for Stroke and Dementia Research (ISD), Feodor-Lynen-Straße 17, Munich 81377, Germany; German Center for Cardiovascular Research (DZHK), Partner Site Munich Heart Alliance, Pettenkoferstraße 8a/9, Munich 81377, Germany; Laboratory of Experimental Cardiology, Department of Cardiology, University Medical Center Utrecht, University Utrecht, Heidelberglaan 100, 3584 CX Utrecht, The Netherlands; Central Diagnostic Laboratory, Division Laboratories, Pharmacy, and Biomedical Genetics, University Medical Center Utrecht, University Utrecht, Heidelberglaan 100, 3584 CX Utrecht, The Netherlands; Laboratory of Experimental Cardiology, Department of Cardiology, University Medical Center Utrecht, University Utrecht, Heidelberglaan 100, 3584 CX Utrecht, The Netherlands; Central Diagnostic Laboratory, Division Laboratories, Pharmacy, and Biomedical Genetics, University Medical Center Utrecht, University Utrecht, Heidelberglaan 100, 3584 CX Utrecht, The Netherlands

**Keywords:** Atherosclerosis, Carotid plaque, Amaurosis fugax, Transient ischaemic attack, Multi-omics

## Abstract

**Aims:**

In patients with atherosclerotic internal carotid artery stenosis, the risk of subsequent ischaemic stroke is higher in those presenting with a cerebral transient ischaemic attack (TIA) than in those with amaurosis fugax (AFX; transient monocular blindness). While AFX is associated with more stable histological characteristics, the underlying molecular mechanisms remain unclear. We hypothesized that biological processes and gene expression profiles in carotid plaques of patients with AFX differ from those with cerebral TIA and stroke.

**Methods and results:**

We analysed carotid plaques from 2508 patients undergoing endarterectomy between 2002 and 2022. Bulk RNA sequencing (RNA-seq) was performed on 1088 plaques (136 asymptomatic, 179 AFX, 481 TIA, 292 stroke). Single-cell RNA-seq from 46 previously profiled plaques was used to identify cell-specific transcriptomic signatures. Mass spectrometry-based proteomics (*n* = 189) and targeted plasma proteomics (*n* = 1291) were used to validate the molecular differences. AFX plaques showed fewer lipid-rich cores than those from cerebral TIA [odds ratio (95% CI): 1.41 (1.03–1.94)] or stroke [1.42 (1.01–1.99)]. RNA-seq identified 408 differentially expressed genes (DEGs) between AFX and TIA and 463 between AFX and stroke (FDR < 0.1), while AFX and asymptomatic plaques showed only 10 DEGs. AFX plaques were enriched for vascular smooth muscle cell signatures and extracellular matrix remodelling, while cerebral TIA and stroke plaques exhibited macrophage-driven inflammatory molecular signatures. These transcriptomic differences were confirmed by plaque proteomic analyses. In parallel, circulating proteomic profiling revealed higher levels of inflammatory and thrombo-inflammatory markers in cerebral TIA and stroke compared with AFX.

**Conclusion:**

AFX plaques exhibit stable, SMC-dominant molecular signatures that differ markedly from the macrophage-driven inflammatory profiles of cerebral TIA and stroke plaques. These findings support AFX as a biologically distinct entity with potential implications for risk stratification and personalized treatment.

**Time of primary review: 27 days**

## 1. Introduction

Stenosis of the internal carotid artery accounts for 10–20% of ischaemic strokes.^1–3^ However, in symptomatic patients, the risk of subsequent ischaemic stroke depends not only on the degree and characteristics of the stenosis but also on the presenting symptom. This risk of recurrent stroke is considerably greater in patients presenting with cerebral transient ischaemic attack (TIA) or ischaemic stroke compared to those with transient monocular blindness, also referred to as amaurosis fugax (AFX), or those who are asymptomatic.^4,5^

AFX is traditionally considered a thromboembolic event caused by carotid atherosclerosis, leading to ischaemia of the retinal artery and optic nerve.^6^ However, its precise aetiology often remains unclear, with potential contributions from nonembolic haemodynamic, ocular, neurologic, or idiopathic mechanisms. Notably, visible retinal emboli are detected in only a minority of AFX cases, and ophthalmologic examinations during symptomatic episodes are not routinely performed.^7^ In many cases, this is simply not feasible, as symptoms typically resolve before patients can be examined.^8–10^ These uncertainties raise questions regarding the benefit of CEA in patients with ocular events.^11^

Emerging evidence suggests that plaque phenotype, rather than stenosis severity alone, plays a key role in symptom manifestation. Histopathological studies indicate that AFX patients more frequently exhibit stable, fibrous plaques, akin to those observed in asymptomatic individuals, whereas patients with cerebral TIA are more likely to have lipid-rich, inflammatory plaques.^12,13^ This suggests that differences in plaque composition may contribute to the variation in stroke risk between AFX and cerebral ischaemic events.

In this study, we aim to uncover transcriptomic features that may explain the lower ischaemic stroke risk associated with AFX and provide insights into its underlying pathophysiology. We hypothesize that AFX and cerebral TIA are driven by distinct biological processes and exhibit differential gene expression (DGE) profiles in carotid plaques.

## 2. Methods

### 2.1 Study design and subject selection

The Athero-Express Biobank Study (AE-biobank) is an ongoing longitudinal biobank that started in 2002. It involves patients undergoing carotid endarterectomy (CEA) at the University Medical Center Utrecht or St. Antonius Hospital in the Netherlands. This study collects extensive baseline data, blood samples, and plaque specimens from patients. The criteria for CEA in the study align with those in international guidelines and are assessed by a multidisciplinary vascular team. Experienced surgeons perform plaque removal following standardized protocols, with pre-operative neurological assessments conducted by neurologists. The cohort study design has been previously described in detail.^14,15^ For the present study, all patients were included who underwent CEA between 2002 and 2022. Symptom categories were ‘asymptomatic’, defined as not having cerebrovascular symptoms in the previous 6 months; AFX was defined as only ipsilateral mono-ocular loss of vision with acute onset lasting less than 24 h. AFX was a clinical diagnosis, made by an ophthalmologist or neurologist; cerebral ‘TIA,’ defined as a focal neurologic deficit of acute onset lasting <24 h; and ipsilateral ‘ischemic stroke’. In patients who had more than one event, the most severe one was considered the classifying event. The baseline characteristics were obtained from patients’ records and through standardized questionnaires. This questionnaire contains questions on the history of cardiovascular disease (CVD), risk factors (smoking habits, hypertension, diabetes mellitus), and use of medication. Current tobacco smoking (i.e. including hand-rolled cigarettes, cigars, etc.) was defined as smoking within 1 year prior to admission for CEA and was assessed by questionnaire. Total cholesterol, triglycerides, low and high-density lipoprotein cholesterol, glucose, haemoglobin, and creatinine were assessed before the operation. This study complies with the Declaration of Helsinki, and all participants provided informed consent. The medical ethical committees of both hospitals approved these studies.

### 2.2 Sample handling

Carotid plaque specimens were removed during surgery and immediately processed in the laboratory. An experienced technician identified the culprit lesion, which is defined as the segment with the smallest lumen. Samples were cut transversely into segments of 5 mm. The culprit lesion (the region with the most severe stenosis) was identified, fixed in 4% formaldehyde, embedded in paraffin, and processed for histological examination. Plaque histological features were routinely scored through chemical and immunohistochemical techniques as described below. The rest of the segments were stored at −80°C.

### 2.3 Histological assessment

The assessment was performed according to a standardized protocol, which was described in detail previously.^16^ Cross-sections of the culprit lesion are stained and quantified for each patient at ×40 magnification. Haematoxylin and eosin (H&E) staining was performed for the assessment of calcification, plaque haemorrhage, and picrosirius red for collagen. Plaque haemorrhage was confirmed by stainings for fibrin and/or glycophorin A. Immunohistochemical staining was performed for assessment of macrophages (CD68) and smooth-muscle cells (alpha-actin). The amounts of collagen, calcifications, and plaque haemorrhage were classified as minor or major. The lipid content of the plaque was estimated as a percentage of the total plaque area, with cut-offs at 10 and 40%. Plaques with <10%, 10–40%, and >40% fat were categorized as fibrous, fibro-atheromatous, and atheromatous, respectively. The criteria for scoring of the smooth muscle cells (SMCs) were defined as follows: (i) no or few scattered cells; (ii) minor alpha-actin staining over the entire circumference with absent staining at parts of the circumference of the arterial wall; (iii) moderate: positive cells along the circumference of the luminal border, with locally at least few scattering cells; (iv) heavy: SMC-dominant plaque with cells within the entire cap and also large clusters deep in the lesion.^16^ All histological characteristics were scored and quantified with dedicated intra-observer and inter-observer reproducibility by two independent observers.^17^

### 2.4 Bulk RNA sequencing and DGE analysis

RNA was isolated from carotid artery plaques using in-house standardized protocols. Then, we employed the CEL-seq2 method for library preparation. The methodology captures the 3′ end of polyadenylated RNA species and includes unique molecular identifiers (UMIs), which allow direct counting of unique RNA molecules in each sample. Plaque sample processing for RNA isolation and library preparation has been described in detail previously.^18^ Libraries were sequenced on the Illumina NextSeq 500 (batch 1) and NovaSeq 6000 (batch 2) platform (Utrecht Sequencing Facility). The reads were demultiplexed and aligned to human cDNA reference (GRCh38.p13/ENSEMBL_GENES_108) using the Burrows–Wheeler Aligner (BWA) (0.7.13) by calling ‘bwa aln’ with settings -B 6 -q 0 -n 0.00 -k 2 -l200 -t 6 for R1 and -B 0 -q 0 -n 0.04 -k 2 -l200 -t 6 for R2, ‘bwa sampe’ with settings -N 100. Multiple reads mapping to the same gene with the same UMI (6 bp long) were counted as a single read. We discarded samples (*n* = 46) with fewer than 9000 detected genes from further analysis.

In all DGE analyses, olfactory-related genes were excluded based on a gene list downloaded from BioMart (see Supplementary material online, *Table S1*). DGE analysis was performed using DESeq2^19^ (version 1.36.0) to compare gene expression between AFX individuals and asymptomatic, cerebral TIA, and stroke individuals. RNA-sequencing data were generated in two technical batches, reflecting different library preparation periods. Prior to differential expression analysis, raw count data were batch-corrected for these known technical batches using the sva package,^20^ without inclusion of any biological or clinical covariates in the batch correction model. Visualization of results was performed using the EnhancedVolcano package (version 1.14.0). Spearman correlation was subsequently applied to assess the consistency of significantly differentially expressed genes (DEGs) (FDR < 0.1) before and after adjustment for confounders. Module scores of significant genes were computed using UCell, and their associations with histological features were evaluated using Spearman correlation.

### 2.5 Principal component analysis

For principal component analysis (PCA), the top 500 most significant genes per comparison, ranked by false discovery rate (FDR), were selected from pairwise differential expression analyses. After removal of duplicate genes across comparisons, 1969 unique genes were retained for PCA. Gene expression counts were normalized using the variance stabilizing transformation (VST) prior to analysis. PCA was performed using the prcomp function in R. PCA visualizations were generated using the factoextra package (version 1.0.7). Differences in mean PC1 and PC2 scores between clinical groups were evaluated using Student’s *t*-tests.

To assess the robustness of the observed clustering and to exclude potential bias introduced by gene selection, an additional PCA was also conducted using all expressed genes (*n* = 21 312). For plasma proteomics, PCA was conducted using all 168 proteins quantified in the CVD panel.

### 2.6 Pathway analysis

Gene set enrichment analysis (GSEA) was performed using the **clusterProfiler**^21^ package (version 4.4.4). All 21 312 genes were ranked based on their log2 fold change values derived from DGE analyses between clinical symptoms. These ranked genes were used as input for the GSEA. Biological processes were clustered using the emapplot function with k-means clustering to group similar pathways.

### 2.7 Mass spectrometry proteomics analysis

Proteins were extracted using sequential incubation with 0.5 M sodium chloride (NaCl), 0.1% sodium dodecyl sulfate, and 4 M guanidine hydrochloride (GuHCl).^10^ All extracts were labelled using tandem mass tags and analysed on an Orbitrap Fusion Lumos Tribrid MS for proteomics (Thermo Scientific). A parallel reaction monitoring method was developed on a Q Exactive HF MS (Thermo Scientific). Protein abundances were normalized based on total protein per sample and scaled using log2 transformation for relative protein quantities. The data were then filtered to only include protein data with less than 30% missing values, and any remaining missing values within the data were imputed using KNN-Impute method, described in detail previously.^22^ In the end, 1499 proteins were detected from 189 plaque lesions. The detailed methodology for mass spectrometry procedures and patient selection has been published previously.^23^

### 2.8 Single-cell mapping of molecular cell types in carotid plaques

scRNA-seq data of human atherosclerotic plaques were published previously.^24^ A total of 20 different cell clusters were identified from 46 patients. In this study, we used addModuleScore within Seurat to calculate module scores for genes highly expressed in AFX and cerebral TIA individuals. This module score calculates the average expression levels of the genes differentially expressed by AFX and cerebral TIA on a single-plaque cell level, subtracted by the aggregated expression of both gene sets. Finally, we created violin plots to plot the obtained module scores over the different cell clusters.

### 2.9 Computational cell type deconvolution

The reference-based cell type deconvolution method used in this study to estimate cellular proportions was Scaden (version 1.1.2), using its default settings.^25^ This technique trained a deep neural network on simulated bulk RNA-seq data derived from the scRNA-seq reference dataset. Datasets of the query bulk RNA-seq and reference scRNA-seq data were prepared in accordance with the accepted format of Scaden, including matching the genes present in both datasets to ensure consistency. The main dataset pairs used as an input for Scaden were (1) reference of the AE plaque, which previously published,^24^ and we consolidated into seven major cell types: dendritic cells, endothelial cells, macrophages, mast cells, memory B cells, SMCs, and T/NK cells. (2) reference of Tabula Sapiens (10 major cell types) with query of the AE plaque, ‘a multiple-organ, single-cell transcriptomic atlas of humans’, which was created to enable the standardized comparisons of cell types across tissues and organs. Hardware limitations required reducing the size of the Tabula Sapiens Seurat object. Of the 24 tissues, 14 were selected: blood, bone marrow, fat, heart, kidney, large intestine, liver, lung, lymph node, muscle, pancreas, small intestine, spleen, and vasculature. Out of the available cell types, 71 were consolidated into 10 major cell types. The 10 major cell types included basophil/mast cell, B cell, endothelial cell, erythrocyte, mesenchymal cell/fibroblast, monocyte, neutrophil, platelet, pericyte/SMC, and T/NK cell. To address considerable variations in the estimated cell type proportions between runs, the deconvolution was repeated 10 times for each pair. The average of these runs was taken for further analysis.

### 2.10 Measurement of circulating biomarkers

Circulating plasma proteins were quantified in a subset of patients from the Athero-Express Biobank using a proximity extension assay platform^26^ (Olink Proteomics AB, Uppsala, Sweden), which enables high-throughput multiplex protein detection. A total of 1291 patients were assayed using the Olink Cardiovascular II and Cardiovascular III panels (*n* = 1291 each), and 654 patients were additionally assessed using the Olink Cardiometabolic panel, which measures 78 cardiometabolism-related proteins. These panels were chosen based on their established relevance to cardiovascular pathophysiology. The detailed methodology has been published previously.^18^ Protein levels are reported as Normalized Protein eXpression values on a log_2_ scale, allowing relative quantification across samples. To minimize technical variability, patient samples were randomly assigned to assay plates and processed according to the manufacturer’s standardized protocols.

### 2.11 Linear regression models

Propensity scores reflecting the probability of inclusion in RNA sub-cohorts were estimated using logistic regression models incorporating age, sex, estimated glomerular filtration rate, smoking status, hypertension diagnosis, and medication use. Bulk RNA-seq and extracellular matrix (ECM)-enriched proteomic data derived from the same plaque samples were integrated to identify overlapping DEGs and proteins, and RNA–protein associations were assessed using Spearman’s rank correlation. To enable comparison across molecular cell types with differing baseline composition, cell-type proportions were standardized to *z*-scores (mean = 0, SD = 1). Linear regression was performed with standardized cell type proportion as the outcome and clinical manifestation as the predictor, using AFX as the reference group. Resulting β coefficients represent standardized mean differences in cell-type proportion between AFX and other clinical groups.

### 2.12 Statistical analysis

Statistical analyses of clinical characteristics were performed using the ‘tableone’ package (version 0.13.2). For this study, comparisons in *Table 1* were limited to the AFX and cerebral TIA groups. Data normality was assessed using the Shapiro–Wilk test. Normally distributed continuous variables were compared using one-way ANOVA, while non-normally distributed continuous variables were analysed with the Kruskal–Wallis test. Categorical variables were compared using the chi-squared test (chisq.test).

**Table 1:** Clinical characteristics of the patient cohort

					*P*value		
	Asymptomatic	AFX	Cerebral TIA	Stroke	AFX vs. asympt	AFX vs. cerebral TIA	AFX vs. stroke
No. of patients	338	412	1072	686			
Age (median [IQR])	67.0 [60.0, 73.0]	69.0 [61.0, 75.2]	70.0 [63.0, 77.0]	71.0 [65.0, 78.0]	0.020	0.012	<0.001
Male (%)	253 (74.9)	276 (67.0)	736 (68.7)	479 (69.8)	0.023	0.579	0.361
Systolic mm Hg (median [IQR])	150 [132, 162]	150 [134, 166]	150 [135, 170]	150 [135, 166]	0.720	0.109	0.287
Diastolic mm Hg (median [IQR])	80 [70, 90]	80 [72, 90]	80 [70, 90]	80 [70, 89]	0.319	0.603	0.106
Laboratory							
BMI kg/m^2^ (median [IQR])	26.7 [24.1, 28.6]	26.2 [24.1, 28.9]	25.9 [24.0, 28.6]	25.9 [23.6, 28.3]	0.732	0.768	0.245
HDL mmol/L (median [IQR])	1.09 [0.92, 1.38]	1.18 [0.94, 1.45]	1.14 [0.94, 1.42]	1.10 [0.90, 1.33]	0.145	0.972	0.035
LDL mmol/L (median [IQR])	2.56 [2.00, 3.42]	2.70 [2.10, 3.60]	2.62 [2.09, 3.39]	2.60 [1.90, 3.40]	0.113	0.591	0.013
TG mmol/L (median [IQR])	1.54 [1.07, 2.22]	1.40 [1.00, 2.20]	1.47 [1.09, 2.04]	1.45 [1.04, 2.00]	0.526	0.719	0.277
TC mmol/L (median [IQR])	4.62 [3.80, 5.51]	4.80 [3.91, 5.60]	4.62 [3.90, 5.60]	4.53 [3.70, 5.40]	0.185	0.714	0.004
Creatinine μmol/L (median [IQR])	91.0 [78.0, 109.0]	85.5 [74.0, 101.0]	89.0 [76.0, 103.0]	87.0 [75.5, 105.0]	0.010	0.034	0.030
Homocysteine μmol/L (median [IQR])	12.8 [10.0, 15.8]	11.5 [10.0, 14.9]	12.6 [10.4, 15.5]	12.9 [10.1, 16.4]	0.438	0.187	0.188
Glucose mmol/L (median [IQR])	6.2 [5.4, 7.3]	5.8 [5.3, 6.6]	6.0 [5.3, 7.1]	6.1 [5.5, 7.3]	0.591	0.697	0.394
eGFR mL/min/1.73 m² (mean (SD))	72.7 (20.7)	76.4 (23.5)	73.14 (20.5)	73.57 (22.4)	0.026	0.008	0.046
Degree of stenosis (%)					0.101	0.643	0.179
0–70%	15 ( 4.6)	34 ( 8.4)	101 ( 9.6)	80 (11.9)			
70–90%	167 (50.8)	207 (50.9)	543 (51.7)	323 (47.9)			
>90%	147 (44.7)	166 (40.8)	406 (38.7)	271 (40.2)			
Days from first event to surgery (median [IQR])		27 [15, 62]	31 [14, 73]	27 [13, 66]		1.00	1.00
History							
Current smoker (%)	100 (29.6)	157 (38.4)	345 (32.5)	232 (34.4)	0.260	0.039	0.210
Hypertension (%)	300 (88.8)	337 (81.8)	929 (86.7)	563 (82.1)	0.011	0.020	0.973
DM (%)	69 (20.4)	85 (20.6)	253 (23.6)	175 (25.5)	1.00	0.249	0.077
CAD (%)	133 (39.3)	116 (28.2)	364 (34.0)	183 (26.7)	0.002	0.039	0.636
Drugs							
Hypertension drugs^a^ (%)	278 (82.7)	302 (73.5)	836 (78.3)	482 (70.5)	0.003	0.058	0.317
Anticoagulants (%)	33 (9.8)	53 (13.0)	128 (12.1)	60 (8.8)	0.223	0.705	0.039
Antiplatelet (%)	311 (92.8)	340 (83.1)	943 (88.2)	596 (87.1)	<0.001	0.012	0.082
LLD (%)	28 (8.3)	24 (5.8)	63 (5.9)	29 (4.2)	0.235	1.000	0.294
Statin (%)	283 (84.2)	320 (77.9)	835 (78.2)	534 (78.1)	0.036	0.948	0.995

The distributions were significantly nonnormal for the variables age, SBP, DBP, BMI, and eGFR according to Shapiro–Wilk tests.

BMI, body mass index; CAD, history of myocardial infarction, coronary artery disease, and/or coronary intervention(s); DM, diabetes mellitus; DBP, diastolic blood pressure; eGFR, estimated glomerular filtration rate; HDL, high-density lipoprotein; IQR, interquartile range; LDL, low-density lipoprotein; LLDs, lipid-lowering drugs other than statins; SD, standard deviation; SBP, systolic blood pressure.

^a^Use of one or more antihypertension drugs.

## 3. Results

### 3.1 Patient cohort and histological features

**Figure 1:**
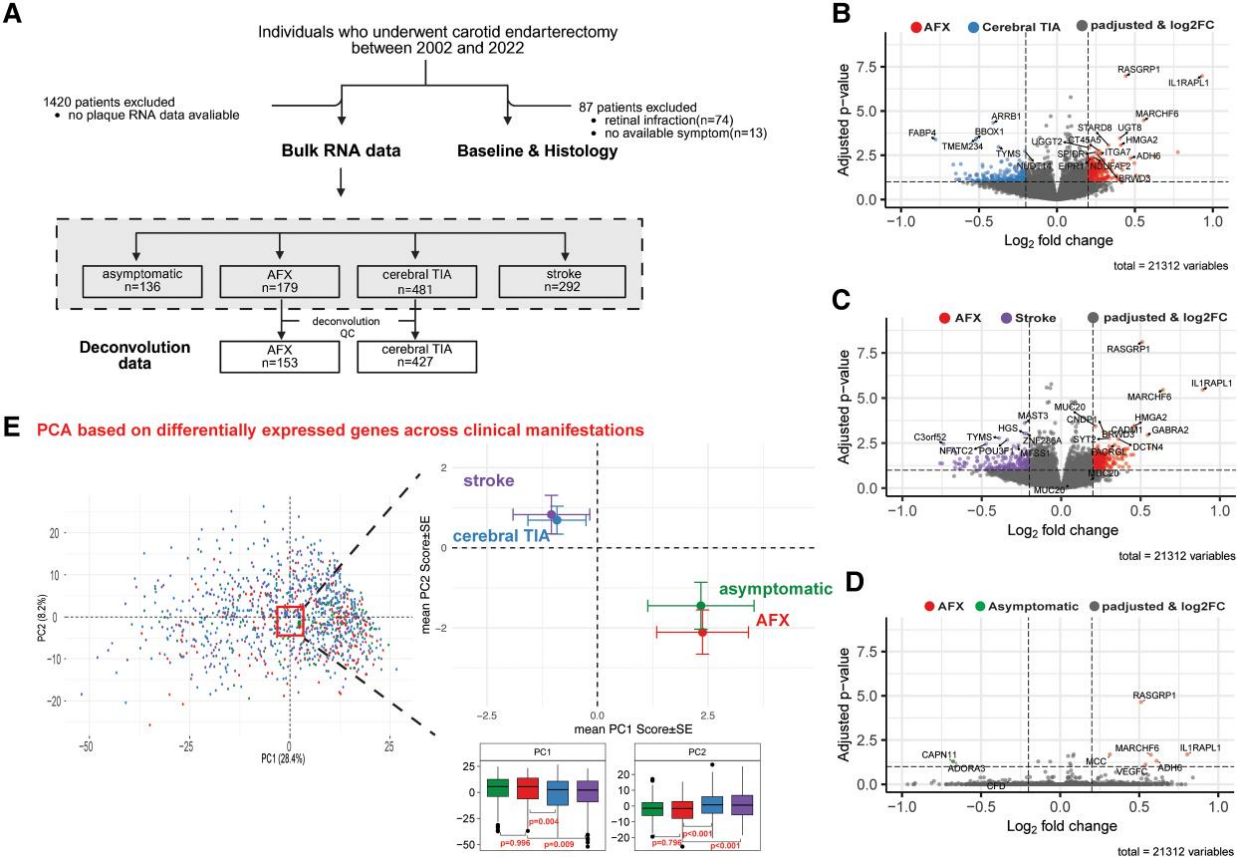
Study cohort and transcriptomic differences between AFX and other clinical presentations. (*A*) Flowchart summarizing the selection process for individuals included in the clinical, histology, transcriptomic, and deconvolution analyses. (*B*) Volcano plot comparing cerebral TIA and AFX plaques. A total of 408 DEGs were identified (226 upregulated in red, 182 downregulated in blue). (*C*) Volcano plot comparing stroke and AFX plaques. A total of 463 DEGs were identified (261 upregulated in red, 202 downregulated in purple). (*D*) Volcano plot of differentially expressed genes (DEGs) between asymptomatic and AFX plaques (false discovery rate [FDR] < 0.1, log₂ fold change > 0.2); red and green denote up- and downregulated genes in AFX, respectively. (*E*) Clustering of patients by clinical manifestation using principal component analysis (PCA) based on 1969 differentially expressed genes. Mean principal component (PC) scores for each clinical group are indicated by red squares. AFX and cerebral TIA demonstrate significant differences in transcriptomic profiles (*P* < 0.001). The mean PC values ± standard error of each group are shown in the right panel. *P*-values were calculated using a *t*-test based on PC scores between AFX and cerebral TIA.

Among the 2508 patients who underwent CEA between 2002 and 2022, 338 (13.4%) were asymptomatic, 412 (16.4%) presented with AFX, 1072 (42.8%) presented with cerebral TIA, and 686 (27.4%) had ischaemic stroke (*Figure 1A*). AFX patients were younger, had lower hypertension prevalence and better renal function, yet displayed higher smoking rates and lower antiplatelet use (*Table 1*). Histologically, plaques from patients with cerebral TIA and stroke had significantly more lipid-rich cores than those from AFX (TIA:30.0% vs. AFX:23.9%; OR 1.39, 95% CI 1.01–1.92; Stroke:30.1% vs. AFX:23.9%; OR 1.53, 95% CI 1.08–2.18) (see Supplementary material online, *Figure S1A*). Representative histological and immunohistochemical images illustrating plaque features across clinical presentation groups are shown in Supplementary material online, *Figure S2*. Three-year post-operative ischaemic stroke and MACE rates did not differ significantly across clinical groups in unadjusted or adjusted Cox regression models (see Supplementary material online, *Figure S1B*–*E*).

### 3.2 Transcriptomic profiling reveals stability-associated signature in AFX plaques

To study the molecular processes in carotid plaques of patients with AFX, we examined bulk RNA-seq data on plaque samples from 136 asymptomatic, 179 AFX, 481 cerebral TIA, and 292 stroke patients from the Athero-Express biobank (*Figure 1A*). The baseline and histological characteristics of this subgroup are presented in Supplementary material online, *Table S2*.

**Figure 2:**
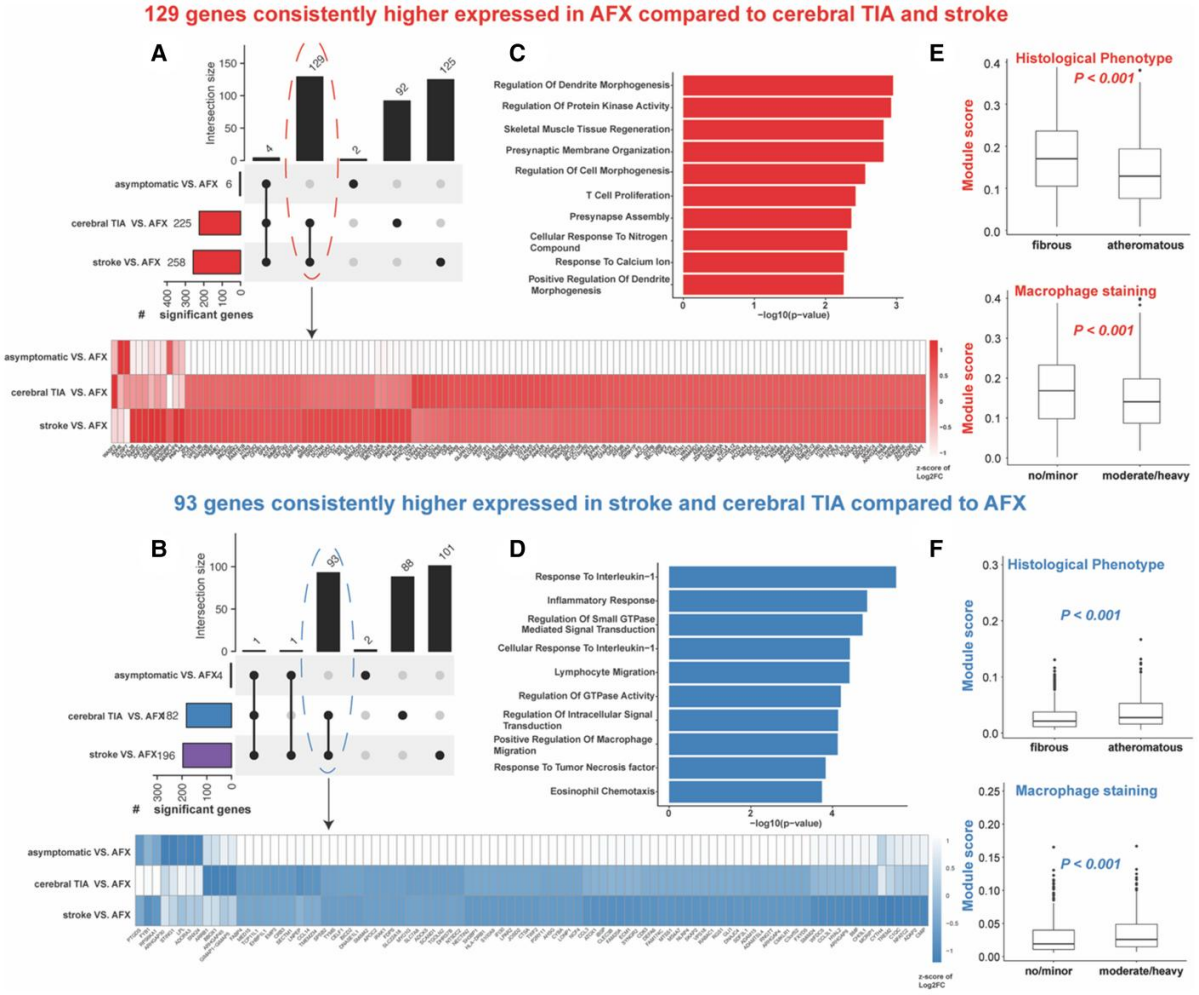
Functional transcriptomic signatures of AFX vs. cerebral TIA and stroke. (*A*) UpSet plot showing the overlap of genes upregulated in AFX across pairwise comparisons. The accompanying heatmap displays z-score normalized expression of the 129 AFX-enriched genes, with panels indicating log_2_ fold change patterns across each comparison. (*B*) UpSet plot and heatmap of the 93 genes upregulated in cerebral TIA/stroke compared with AFX. The heatmap shows z-score normalized expression and log_2_ fold change profiles across pairwise comparisons. (*C*, *D*) Top 10 enriched biological processes for genes consistently higher in AFX (129 genes; *C*) and those consistently higher in cerebral TIA/stroke (93 genes; *D*) based on differential expression derived functional enrichment analysis. (*E*, *F*) Associations between gene expression module scores and plaque histological phenotype and macrophage staining, in AFX and cerebral TIA/stroke. Module scores were derived from the 129 AFX-enriched genes (*E*) and the 93 cerebral TIA/stroke-enriched genes (*F*), and group differences were assessed using ANOVA.

Among 21 312 expressed genes, DGE analysis revealed 408 significantly different genes between AFX and cerebral TIA (226 upregulated, 182 downregulated) and 463 between AFX and stroke (261 upregulated, 202 downregulated) at an FDR < 0.1 and log_2_ fold change > 0.2 (*Figure 1B* and *C*; Supplementary material online, *Tables S3* and *S4*). These gene expression patterns were robust, with strong concordance between crude and multivariable adjusted models Spearman correlation coefficients of 0.94 for AFX vs. TIA and 0.97, for AFX vs. stroke (see Supplementary material online, *Figure S3A*). In contrast, AFX and asymptomatic plaques showed minimal transcriptomic differences, with only 10 DEGs (4 upregulated, 6 downregulated) (*Figure 1D*; Supplementary material online, *Tables S5*). PCA showed clustering of AFX profiles with asymptomatic plaques, distinctly separated from TIA and stroke groups (*Figure 1E*). This clustering pattern remained consistent when PCA was repeated using all expressed genes, confirming the robustness of the observation (see Supplementary material online, *Figure S3B*). The AFX and TIA split remained significant in males (*P* = 0.001), not females (*P* = 0.181) (see Supplementary material online, *Figure S4*). Gene subset analysis identified 129 AFX higher expression genes linked to vascular smooth muscle cell (VSMC) adaptation and extracellular matrix pathways, and 93 TIA/stroke higher expression genes enriched in inflammatory responses (*Figure 2A–D*). GSEA showed AFX’s enrichment in extracellular matrix/mesenchyme development/vasoconstriction pathways, while TIA and stroke lesions were more inflammation-oriented (see Supplementary material online, *Figures S5–S7*; *Tables S6–S8*).

To better understand how gene expression patterns relate to plaque pathology, we examined the association between transcriptomic module scores and key histological features of plaque vulnerability. AFX-associated gene module activity was significantly higher in fibrous plaques and inversely associated with macrophage content (*P* < 0.001). Conversely, TIA and stroke-associated gene module activity correlated strongly with atheromatous plaque phenotype and increased macrophage infiltration (*P* < 0.001) (*Figure 2E* and *F*).

### 3.3 ECM proteome profiling of carotid plaques mirrors transcriptomic changes between AFX and cerebral TIA

To validate transcriptomic findings at the protein level and to explore functional differences between AFX and cerebral TIA plaques, we analysed plaque proteins using mass spectrometry. Plaque proteomic profiling was conducted across a cohort of 189 patient subgroups, including 28 asymptomatic, 41 AFX, 84 TIA, and 36 stroke patients (see Supplementary material online, *Table S9*).

**Figure 3:**
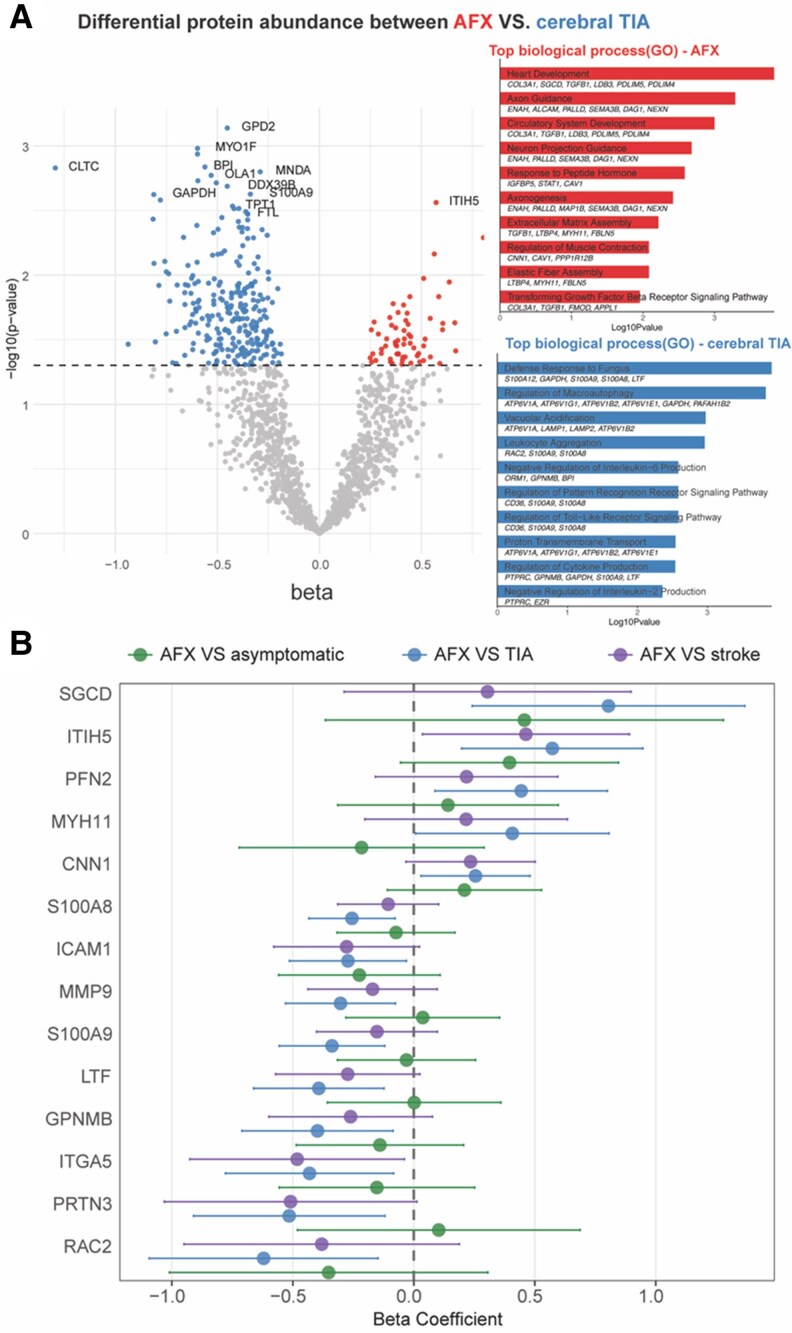
Plaque proteomic profiling reveals extracellular matrix–related differences between amaurosis fugax (AFX) and cerebral transient ischaemic attack (TIA). (*A*) Volcano plot showing differential protein expression (*n* = 1499 proteins) in carotid plaques from AFX vs. cerebral TIA. Proteins reaching the nominal significance threshold (dashed line, *P* = 0.05) are highlighted in red for AFX-enriched proteins and blue for TIA enriched proteins. (*B*) Forest plot showing relative protein abundance levels across clinical comparisons: AFX vs. stroke (purple), AFX vs. TIA (blue), and AFX vs. asymptomatic plaques (green). Protein and clinical manifestations associations were assessed using linear regression models adjusted for age and sex, with effect sizes displayed as regression coefficients and corresponding confidence intervals.

Plaque proteomic profiling revealed 322 nominally differentially abundant proteins between AFX and cerebral TIA. Functional enrichment analysis revealed that proteins enriched in AFX plaques were predominantly associated with extracellular matrix organization and smooth muscle structure, whereas proteins elevated in cerebral TIA plaques mapped to inflammatory and immune-related processes (*Figure 3A*). Similar enrichment of fibrous-associated pathways was observed when comparing AFX with stroke plaques, whereas no such differences were detected between AFX and asymptomatic plaques (see Supplementary material online, *Figure S8A* and *B*). Among the top proteins, inflammatory proteins S100A8 and S100A9 were elevated in cerebral TIA plaques. The effect sizes of these markers were comparable when contrasting AFX with cerebral TIA and AFX with stroke, whereas differences between AFX and asymptomatic plaques were minimal; however, fibrous matrix-associated proteins such as CNN1 and ITIH5 were more abundant in AFX plaques (*Figure 3B*).

Fibrosis-related proteins showed predominantly positive RNA and protein correlations (see Supplementary material online, *Figure S8C*). Concordance analysis further identified 10 gene/protein pairs with consistently higher levels in cerebral TIA compared with AFX (see Supplementary material online, *Figure S8D*; Supplementary material online, *Table S10*).

### 3.4 Smooth muscle cells dominate the stable transcriptomic signature in AFX plaques, contrasting with macrophage-driven inflammation in cerebral TIA plaques

**Figure 4:**
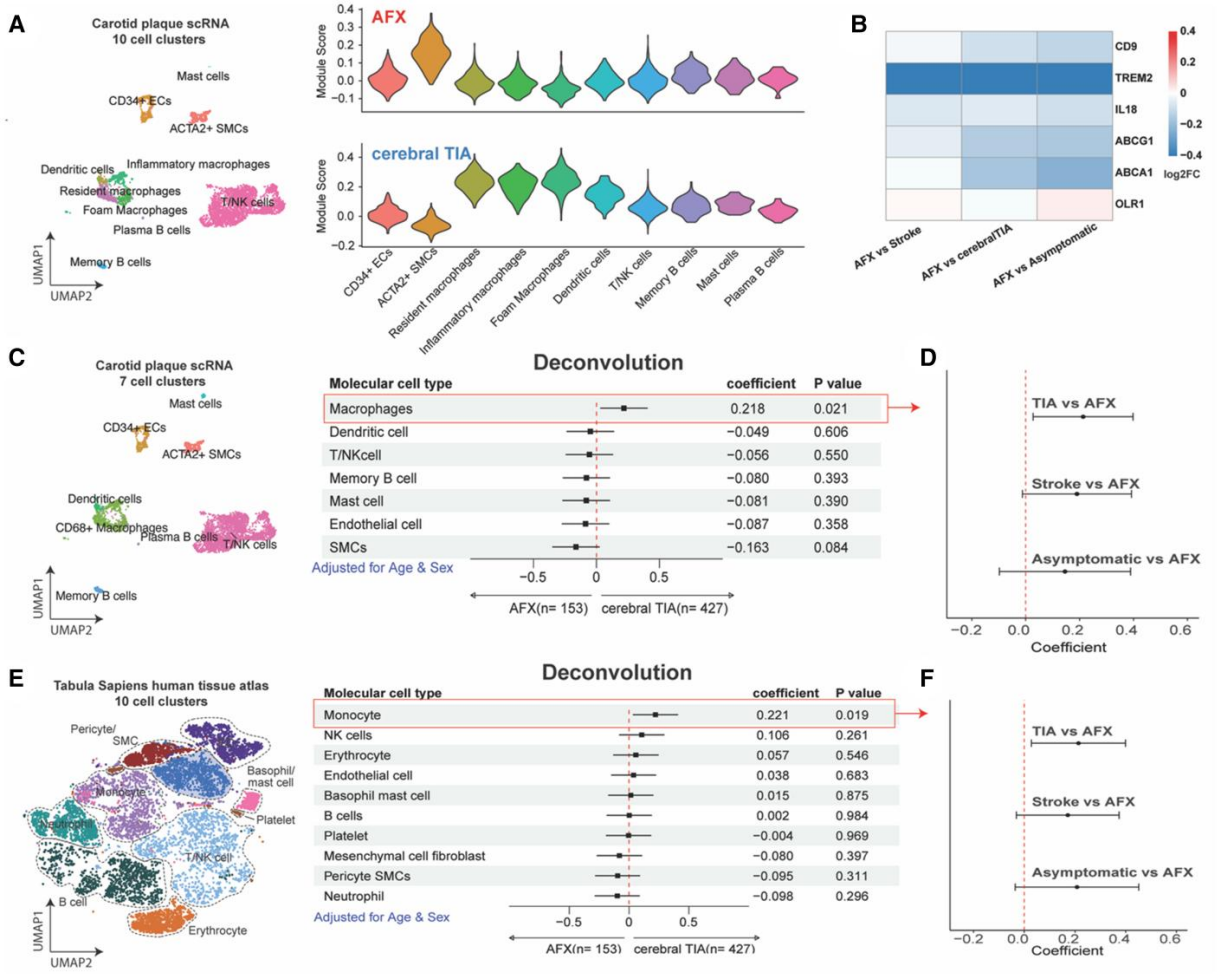
Molecular and cellular characteristics of individuals with AFX and TIA. (*A*) UMAP plot depicting the 10 cell clusters merged by transcriptomics from 46 carotid lesion samples^1^ and Violin plot showing the module scores of genes that are highly expressed in AFX or cerebral TIA carotid lesions in single-cell clusters detected via single-cell RNA sequencing of human atherosclerotic plaques. (*B*) Heatmap of foam cell-associated marker genes expression across patient groups. (*C*) Estimated plaque cell-type proportions across clinical groups derived from deconvolution of bulk RNA sequencing data using a carotid plaque single-cell reference, adjusted for age and sex. (*D*) Associations between estimated macrophage proportions and clinical groups, expressed as z-scores from linear regression models adjusted for age and sex, with AFX used as the reference group. (*E*) Estimated plaque cell-type proportions across clinical groups derived from deconvolution using the Tabula Sapiens multitissue reference, adjusted for age and sex. (*F*) Associations between estimated monocyte proportions and clinical groups, expressed as *z*-scores from linear regression models adjusted for age and sex, with AFX as the reference group.

Because the largest molecular differences were observed between AFX and cerebral TIA plaques, we next sought to identify the contributing cell types by integrating bulk and single-cell transcriptomic data. We analysed single-cell transcriptomic datasets from 46 human carotid lesions previously published,^24^ comprising 10 major cell clusters (*Figure 4A*). AFX-associated gene module scores were highest in ACTA2-positive SMCs (mean module score 0.83), whereas cerebral TIA-associated gene module scores were highest in inflammatory and foam macrophage clusters (mean module scores 0.20 and 0.24, respectively). Notably, several foam-cell marker genes, including TREM2 (FDR < 0.1), were more highly expressed in cerebral TIA and stroke plaques (*Figure 4B*). Module scores across cell types were comparable between female and male patients (see Supplementary material online, *Figure S9A*).

To quantify cell-type composition in bulk RNA sequencing samples, we conducted deconvolution analysis in 153 AFX and 427 cerebral TIA individuals, using both a carotid plaque single-cell and the Tabula Sapiens multi-tissue reference.^27^ Cerebral TIA plaques showed a significantly higher proportion of macrophages (β = 0.22, *P* = 0.021). This pattern was consistent when comparing AFX with asymptomatic and stroke plaques, although statistical significance was reached only for the cerebral TIA comparison (*Figure 4C* and *D*). Similar results were obtained using the Tabula Sapiens reference, where monocyte proportions were significantly elevated in cerebral TIA plaques (β = 0.22, *P* = 0.019) and lowest in AFX plaques (*Figure 4E* and *F*). Sex-stratified analyses indicated that these increases were more pronounced in male patients, while effects in female patients were directionally consistent (see Supplementary material online, *Figure S9B*).

### 3.5 Circulating proteomic signatures distinguish AFX from cerebral TIA and stroke

**Figure 5:**
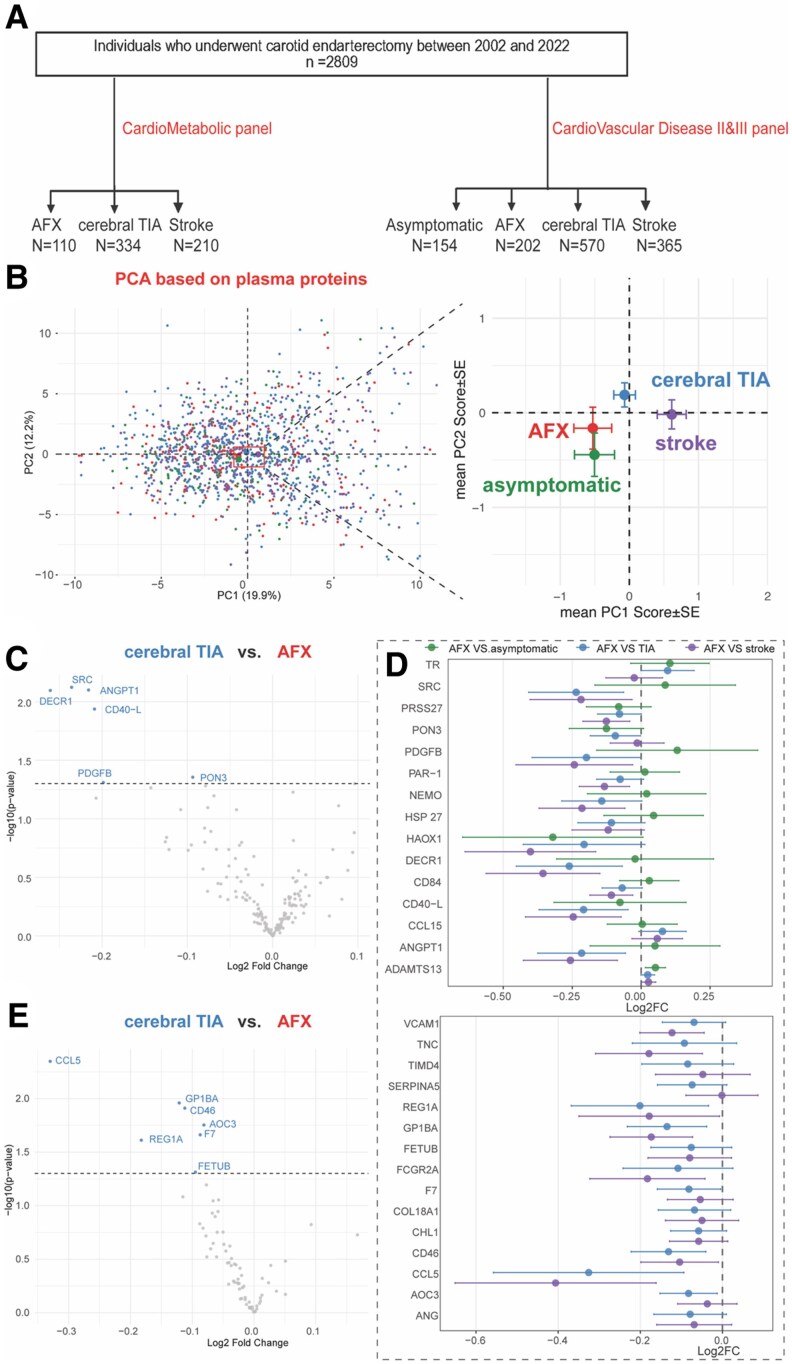
Plasma proteomic profiling distinguishes AFX from cerebral TIA. (*A*) Flow diagram of patients profiled using Olink Cardiovascular II and III (CVD) and cardiometabolic (CM) panels. (*B*) Principal component analysis (PCA) of proteins (*n* = 168) quantified in the CVD panel, illustrating clustering according to clinical presentation. Red squares indicate group mean PCA scores ± standard error. (*C*) Volcano plot showing differential abundance of CVD panel proteins comparing AFX with cerebral TIA. (*D*) Forest plots displaying relative protein abundance across clinical comparisons (AFX vs. asymptomatic, AFX vs. cerebral TIA, and AFX vs. stroke) for CVD (upper panel) and CM (lower panel) proteins. (*E*) Volcano plot showing differential abundance of CM panel proteins comparing AFX with cerebral TIA. For panels *C*–*E*, associations between plasma protein levels and clinical presentation were assessed using linear regression models adjusted for age, sex, estimated glomerular filtration rate (eGFR), smoking status, hypertension, and antiplatelet use. Effect sizes are presented as log_2_ fold change with corresponding standard errors and 95% confidence intervals.

To determine whether plaque-associated molecular differences were reflected systemically, we analysed plasma proteomic data using Olink CVD and cardiometabolic (CM) panels (*Figure 5A*; Supplementary material online, *Table S9*).

PCA based on 168 CVD panel proteins showed that AFX clustered closely with asymptomatic individuals while forming a distinct cluster from cerebral TIA and stroke patients (mean PC score difference, *P* = 0.146 for AFX vs. asymptomatic; *P* < 0.001 for AFX vs. cerebral TIA/stroke; *Figure 5B*). In the CVD panel, 7 of 168 proteins showed nominal differences between AFX and cerebral TIA after adjustment for clinical confounders (*Figure 5C*). Inflammatory proteins such as CD40L, CCL15, and SRC were elevated in cerebral TIA, whereas ADAMTS13 showed higher levels in AFX patients. Effect sizes for these proteins were comparable when contrasting stroke with AFX, while differences between asymptomatic individuals and AFX were minimal (*Figure 5D* upper panel).

Consistent findings were observed in the CM panel, where circulating CCL5 and GP1BA levels were significantly higher in cerebral TIA and similarly elevated in stroke patients compared with AFX (*Figure 5C* lower panel; *Figure 5D*). Plaque and plasma proteomics overlap is illustrated in Supplementary material online, *Figure S10A*. Across panels, sex-stratified analyses demonstrated consistent directions of effect in males and females (see Supplementary material online, *Figure S10B* and *C*).

## 4. Discussion

In this study of plaques dissected from patients undergoing CEA, we demonstrate that those from patients with AFX exhibit transcriptomic features that represent more stable fibrous plaque compared to those from patients with a cerebral TIA or ischaemic stroke. Notably, AFX lesions share significant similarities with asymptomatic plaques, suggesting a lower risk of plaque instability and inflammation. Histological and genetic analyses support these findings. To our knowledge, this is the first study to provide a comprehensive biological characterization of carotid plaques in patients with AFX.

Our transcriptomic analysis revealed that AFX lesions have a more stable molecular profile compared to TIA and stroke lesions, aligning with our histological findings and prior histological studies^13^ of carotid plaques. Carotid plaques from patients with AFX exhibited a higher presence of activated ACTA2^+^ SMCs, indicating a more stable plaque phenotype or increased fibrous plaque content at the end stage. By contrast, cerebral TIA lesions displayed a greater abundance of fat content and macrophage staining, which are considered key markers of plaque vulnerability and may contribute to plaque rupture.^28–31^ Additionally, we identified DGE patterns associated with clinical manifestation. For instance, *FABP4* and *TREM2*, known to be involved in foam cell formation and macrophage-driven inflammation, were highly expressed in cerebral TIA and stroke plaques, reinforcing their role in plaque destabilization. Similarly, *S100A8*/*S100A9*, key mediators of inflammatory responses in atherosclerosis, were upregulated in patients with cerebral TIA or stroke compared to those with AFX.^32–35^

Furthermore, genes related to VSMCs and endothelial cells were more highly expressed in AFX patients, suggesting active vascular repair and plaque stabilization in this group. In contrast, TIA and stroke patients exhibited stronger inflammatory and immune responses, which are known to drive plaque instability and increase the risk of recurrent ischaemic events. Sex-stratified analysis further reinforced the distinction between AFX and cerebral TIA or stroke based on transcriptomic features. However, while AFX lesions in males clustered distinctly from TIA and stroke, AFX lesions in females exhibited a less pronounced separation. This could be attributed to the overall greater plaque stability typically observed in female patients, which may obscure transcriptomic differences within this subgroup.^36^

Striking was the observation that in the clustering analyses, the molecular profiles of AFX lesions closely resemble asymptomatic plaques, distinct from those of cerebral TIA or stroke patients. The stable molecular features observed in both AFX and asymptomatic carotid plaques were further confirmed by transcriptome-defined cell composition. Notably, cerebral TIA plaques exhibited a higher presence of macrophage- or monocyte-associated molecular signatures (coefficient 0.21 and 0.22, respectively), depending on the reference tissues used.

These molecular and histological observations align with ultrasound imaging findings, which indicate that AFX plaques are more homogeneous and echolucent compared to plaques in cerebral TIA and stroke. Interestingly, despite transcriptomic similarities between AFX and asymptomatic plaques, ultrasound imaging revealed distinct morphological differences: asymptomatic plaques appeared more hyperechoic, whereas AFX plaques exhibited a more echolucent profile.^37,38^ The relative stability of AFX plaques may also explain the lower perioperative risk observed in ocular ischaemic patients undergoing carotid artery interventions compared to those with cerebral TIA or stroke.^39,40^ Additionally, PET-CT studies using ^18^F-sodium fluoride^41^ have demonstrated increased tracer uptake in vulnerable plaques and higher aortic activity in patients with ischaemic stroke, reflecting active microcalcification and atheromatous disease activity. Although imaging data were not available in the present cohort, the inflammatory and macrophage-rich molecular signatures observed in cerebral TIA and stroke plaques are consistent with patterns described in vulnerability-oriented imaging studies, highlighting the potential value of future integration of molecular profiling with advanced PET or CTA imaging.

These distinct histological, transcriptomic, and protein features suggest that the underlying mechanisms of AFX may differ from those of cerebral TIA and stroke. One potential explanation is retinal artery spasm, which has been implicated in transient monocular blindness in previous studies.^7,42,43^ Like vasospasm observed in other coronary vascular disorders, retinal artery spasms may result from both endothelial and non-endothelial dysfunction in the microvasculature,^44–46^ leading to temporary vision loss without overt plaque rupture. In line with this hypothesis, our plasma proteomics analysis demonstrated that patients with cerebral TIA and stroke had elevated levels of inflammatory and endothelial activation markers, including VCAM1, CCL5, and GP1BA, consistent with a more vulnerable plaque phenotype. These proteins have been previously associated with leucocyte recruitment, endothelial dysfunction, and pro-thrombotic signalling pathways, all of which contribute to atherosclerotic progression and embolic risk.^47,48^ Furthermore, DECR1, a mitochondrial enzyme implicated in lipid peroxidation and cardiovascular stress responses, was also elevated in cerebral ischaemic events and has been associated with major adverse cardiovascular outcomes in earlier carotid cohort studies.^49^ In contrast, the lower expression of these markers in AFX supports a more stable biological profile, both locally within the plaque and systemically.

Overall, our findings underscore the need for further research to optimize the management of AFX patients. Current treatment guidelines often group AFX with other symptomatic carotid events, potentially overlooking the distinct plaque stability and pathophysiology that set AFX apart. Tailored treatment strategies specific to AFX patients could improve clinical outcomes and reduce unnecessary interventions.

This study is limited by using patients with high-degree stenosis of carotid lesions who underwent CEA. As such, we could not investigate the mechanisms driving the development and progression of carotid plaques at earlier stages of the disease. Additionally, the diagnosis of AFX and TIA is largely based on clinical history, with a significant reliance on patient-reported symptoms. Despite the involvement of specialists such as neurologists and ophthalmologists and multidisciplinary panels, the diagnostic process remains inherently subjective. This subjectivity introduces variability and contributes to diagnostic uncertainty, which may affect the interpretation of symptom-specific findings. Finally, the time interval between the clinical event and plaque collection represents an additional limitation, as inflammatory resolution and vascular remodelling may occur following plaque rupture; although the time from first event to surgery did not differ significantly between symptomatic groups in our cohort, ongoing effect of plaque healing cannot be excluded. In conclusion, our study highlights the stable transcriptomic and histological characteristics of carotid plaques in patients with AFX, which align more closely with asymptomatic carotid plaques than with lesions of patients with cerebral TIA or ischaemic stroke.

Translational perspective From the translational perspective, these findings challenge the current classification of AFX as part of the broader symptomatic carotid disease group and underscore the need for further clinical trials to evaluate the clinical benefit of medical treatment and revascularization strategies tailored specifically to AFX patients.

## Supplementary Material

cvag116_Supplementary_Data

## Data Availability

Bulk RNA-seq data and associated clinical metadata from part of the Athero-Express (AE) cohort are available via DataverseNL (DOIs: https://doi.org/10.34894/D1MDKL, https://doi.org/10.34894/4IKE3T). Due to data governance and privacy regulations, access to private patient data is controlled and can be requested through DataverseNL. All analysis code used in this study is publicly available at https://github.com/LTinthesky/carotid-plaque-omics-AFX-TIA/tree/main/code, including R scripts for the main bulk RNA-seq analyses.
