# Supplementary material for: Gene expression profiles of carotid plaques differ between patients with amaurosis fugax and those with cerebral transient ischaemic attack or stroke
Source: Cardiovasc Res. 2026 Jun 2;122(11):1443–54. doi: 10.1093/cvr/cvag116 (PMC13473970; doi:10.1093/cvr/cvag116)
Supplement: Supplementary file 1 [file cvag116_supplementary_data.zip › Supplementary figure_CVR2025-1577.docx]

**Supplementary Figure 1. Histological characteristics and postoperative outcomes across clinical presentation groups.** A.Odds ratios of histological features across asymptomatic, cerebral TIA, and stroke lesions, using amaurosis fugax (AFX) lesions as the reference group. The analysis was performed with a generalized linear model (GLM) adjusted for age and sex. Key histological features include intraplaque hemorrhage (IPH) and smooth muscle cells (SMCs).B Kaplan–Meier curve of ischemic stroke-free survival within 3 years after surgery. C.Kaplan–Meier curve of major adverse cardiovascular event (MACE)-free survival within 3 years after surgery.D Hazard ratios (HRs) for ischemic stroke-free survival within 3 years after surgery.E Hazard ratios (HRs) for MACE within 3 years after surgery. AFX, amaurosis fugax; TIA, transient ischemic attack; IPH, intraplaque hemorrhage; SMCs, smooth muscle cells; MACE, major adverse cardiovascular event.

**Supplementary Figure 2. Histological characteristics of carotid plaques across clinical manifestations.**

Representative histological stainings of carotid plaques from asymptomatic (A), amaurosis fugax (B) cerebral transient ischemic attack (TIA) (C), and stroke (D) patients. Hematoxylin eosin (H&E) and Sirius Red staining demonstrating higher collagen content and a more fibrous plaque composition in asymptomatic and AFX plaques. Immunohistochemical staining for smooth muscle actin (SMA) and CD68 showing increased smooth muscle cell rich fibrous tissue in asymptomatic and AFX plaques, whereas cerebral TIA and stroke plaques display greater macrophage infiltration and a more atheromatous phenotype.

**Supplementary Figure 3. Robustness of transcriptomic profiling after adjustment for clinical confounders.**

**A.** Correlation of significantly differentially expressed genes (false discovery rate < 0.1 and |log₂ fold change| > 0.2) between univariable and multivariable differential expression analyses comparing amaurosis fugax (AFX) with cerebral transient ischemic attack (TIA) and stroke. Correlations were assessed using Pearson’s correlation coefficient. Multivariable models were adjusted for age, sex, estimated glomerular filtration rate (eGFR), smoking status, hypertension, and antiplatelet use. **B.** Principal component analysis (PCA) based on all 21312 expressed genes, demonstrating clustering of samples according to clinical manifestations. Mean PC1 and PC2 scores for each clinical group are shown in the right panel.

**Supplementary Figure 4. Clustering of clinical manifestation groups based on transcriptomic data, stratified by sex.**

A**.** Clustering of female patients based on transcriptomic features, with mean principal component (PC) scores highlighted by red squares. AFX and cerebral TIA exhibit distinct transcriptomic profiles (p = 0.18). The mean PC values ± standard error for each group are shown in the right panel. B**.** Clustering of male patients based on transcriptomic features, with mean PC scores highlighted by red squares. AFX and cerebral TIA demonstrate significant differences in transcriptomic profiles (p = 0.001). The mean PC values ± standard error for each group are shown in the right panel. *P*-values were calculated using a t-test based on mean PC scores between AFX and cerebral TIA.AFX, amaurosis fugax; TIA, transient ischemic attack.

**Supplementary Figure 5. Gene Set Enrichment Analysis Between AFX and Asymptomatic Lesions**. This figure illustrates the results of gene set enrichment analysis comparing AFX and asymptomatic lesions. **A.** Top biological processes enriched in AFX lesions. **B.** Top biological processes enriched in asymptomatic lesions. All biological processes are grouped into five clusters based on similar biological functions, with each cluster represented by a distinct colour. AFX : amaurosis fugax.

**Supplementary Figure 6. Gene Set Enrichment Analysis Between AFX and Cerebral TIA Lesions**. This figure illustrates the results of gene set enrichment analysis comparing AFX and cerebral TIA lesions. **A.** Top biological processes enriched in AFX lesions. **B.** Top biological processes enriched in cerebral TIA lesions. All biological processes are grouped into five clusters based on similar biological functions, with each cluster represented by a distinct colour. AFX : amaurosis fugax. TIA : transient ischemic attacks.

**Supplementary Figure 7. Gene Set Enrichment Analysis Between AFX and Stroke Lesions**. This figure illustrates the results of gene set enrichment analysis comparing AFX and stroke lesions. **A.** Top biological processes enriched in AFX lesions. **B.** Top biological processes enriched in stroke lesions. All biological processes are grouped into five clusters based on similar biological functions, with each cluster represented by a distinct colour. AFX : amaurosis fugax.

**Supplementary Figure 8. Gene and protein correlations and differential protein profiles in amaurosis fugax (AFX) and asymptomatic/stroke.**

A. Volcano plot showing differential protein abundance between AFX and asymptomatic plaques. B. Volcano plot showing differential protein abundance between AFX and stroke plaques. In panels A and B, Gene Ontology enrichment analysis was performed for top 100 proteins and significant different proteins (p < 0.05) are highlighted in red for AFX, green for asymptomatic, and purple for stroke. C. Spearman correlation analysis between carotid plaque gene expression and protein abundance in AFX and cerebral TIA; Gene Ontology (GO) enrichment analysis was performed for proteins showing significant positive correlations with transcript levels (FDR < 0.05, correlation coefficient > 0). D. Overlap of significantly differentially expressed plaque genes and significantly altered proteins between AFX and cerebral TIA.

**Supplementary Figure 9. Cell type composition differences between AFX and cerebral TIA stratified by sex.**

A. Sex-specific differences in cell type module scores between amaurosis fugax (AFX) and cerebral TIA patients from single-cell RNA-seq data (females: n=20; males: n=26). Module scores quantify expression of gene signatures for 10 major cell populations derived from published carotid plaque single-cell atlases. **B.** Cell composition profiles from bulk RNA-seq deconvolution showing 7 cell types (using bulk RNA reference) and 10 cell clusters (using Tabula Sapiens reference). Effect estimates represent differences in cell proportions (cerebral TIA vs AFX) from linear regression models. Points show effect estimates; error bars indicate 95% confidence intervals. AFX, amaurosis fugax; TIA, transient ischemic attack.

**Supplementary Figure 10. Sex-stratified analysis of circulating proteins comparing AFX and cerebral TIA.**

**A.** Venn diagram summarizing overlap between differentially abundant proteins in plaque ECM (n=1499) and plasma proteomics(n=168) across clinical comparisons: no overlap in asymptomatic vs AFX (30 plaque, 22 plasma) or stroke vs AFX (63 plaque, 39 plasma), and limited overlap in cerebral TIA vs AFX, where DECR1 and SRC were consistently more abundant in cerebral TIA (p < 0.05). **B.** Differential abundance of circulating proteins in males from the CVD and CM panel across clinical symptom comparisons. **C.** Differential abundance of circulating proteins in females from the CVD and CM panel across clinical symptom comparisons. Effect sizes are shown for comparisons between asymptomatic and amaurosis fugax (AFX) (green), AFX and cerebral transient ischemic attack (TIA) (blue), and AFX and stroke (purple). Differential protein abundance was assessed using limma models adjusted for age, sex (where applicable), estimated glomerular filtration rate (eGFR), smoking status, hypertension, and antiplatelet use. Proteins marked with an asterisk (*) indicate statistically significant differences in the comparison between AFX and cerebral TIA.
